# Neural Interactions in a Spatially-Distributed Cortical Network During Perceptual Decision-Making

**DOI:** 10.3389/fnbeh.2019.00220

**Published:** 2019-09-24

**Authors:** Vladimir A. Maksimenko, Nikita S. Frolov, Alexander E. Hramov, Anastasia E. Runnova, Vadim V. Grubov, Jürgen Kurths, Alexander N. Pisarchik

**Affiliations:** ^1^Neuroscience and Cognitive Technology Laboratory, Center for Technologies in Robotics and Mechatronics Components, Innopolis University, Innopolis, Russia; ^2^Research Domain IV “Complexity Science”, Potsdam Institute for Climate Impact Research, Potsdam, Germany; ^3^Department of Physics, Humboldt University, Berlin, Germany; ^4^Faculty of Biology, Saratov State University, Saratov, Russia; ^5^Center for Biomedical Technology, Technical University of Madrid, Madrid, Spain

**Keywords:** perceptual decision-making task, reaction time, behavioral response fluctuations, cortical network reorganization, functional brain network

## Abstract

Behavioral experiments evidence that attention is not maintained at a constant level, but fluctuates with time. Recent studies associate such fluctuations with dynamics of attention-related cortical networks, however the exact mechanism remains unclear. To address this issue, we consider functional neuronal interactions during the accomplishment of a reaction time (RT) task which requires sustained attention. The participants are subjected to a binary classification of a large number of presented ambiguous visual stimuli with different degrees of ambiguity. Generally, high ambiguity causes high RT and vice versa. However, we demonstrate that RT fluctuates even when the stimulus ambiguity remains unchanged. The analysis of neuronal activity reveals that the subject's behavioral response is preceded by the formation of a distributed functional network in the β-frequency band. This network is characterized by high connectivity in the frontal cortex and supposed to subserve a decision-making process. We show that neither the network structure nor the duration of its formation depend on RT and stimulus ambiguity. In turn, RT is related to the moment of time when the β-band functional network emerges. We hypothesize that RT is affected by the processes preceding the decision-making stage, e.g., encoding visual sensory information and extracting decision-relevant features from raw sensory information.

## 1. Introduction

When performing a task implying visual information processing and decision-making (perceptual decision-making task), the brain dynamically adjusts the structure of its functional network so as to maintain an optimal behavioral performance under the increasing cognitive demand (Parks and Madden, 2013; Davison et al., 2015; Shine and Poldrack, 2018). Modern neurophysiological studies emphasize the leading role of the brain functional connectivity in human cognition and behavioral performance (Smith, 2016). According to functional magnetic resonance imaging (fMRI) studies, the whole-brain network activity is generated through the interaction of multiple functional subnetworks during either a resting state or task accomplishing. These functional subnetworks include a dorsal attention network, a frontoparietal network, an executive control network, a default mode network, etc. (Van Den Heuvel and Pol, 2010). Although functional networks have different anatomical locations, they interact with each other and overlap during task accomplishing (Xu et al., 2013).

In their recent work, Rosenberg et al. (2016) demonstrated that brain functional connectivity restored from fMRI during a resting state can predict the subject's ability to maintain sustained attention during demanding tasks. Next, Li et al. (2016) showed that a fatigue-related decrease in behavioral performance during a long-term cognitive task is accompanied by topology reshaping of the functional brain connectivity network. Namely, brain regions become more segregated and their communication is less efficient under a fatigue state. Furthermore, Xu et al. (2014) indicated that high and low attention demands engage a different functional network architecture. In particular, the left frontoparietal network is mostly implicated during a low attention load, while the dorsal attentional network is involved in tasks that require high attention. In addition, Finc et al. (2017) highlighted that an increase in cognitive demands results in a decrease in network modularity. In this case, the default mode network enhances its connectivity with other networks, while the connectivity inside the network itself decreases. Finally, it was shown that along with slow fatigue-related and demand-related changes there are spontaneous fluctuations in functional connectivity that affect behavioral performance. In this respect, Kucyi et al. (2017) evidenced that when the attention level fluctuates during a long-term attention task, different parts of the attention-related network (dorsal-attention and default-mode networks) exhibit antiphase changes in functional connectivity. In addition, the fMRI study allowed Elton and Gao (2015) to reveal the relationship between task-related changes in functional connectivity fluctuations and task performance. Recently, slow rhythmic oscillations of sustained attention were detected during a prolonged cognitive load (Helfrich et al., 2018; Maksimenko et al., 2018a, 2019).

Along with fMRI, functional network connectivity can effectively be restored from the signals of electrical brain activity, electroencephalograms (EEG), recorded by non-invasive electrodes. EEG signals are composed of various rhythms of neural activity in different frequency bands, e.g., δ-band (1–5 Hz), θ-band (5–8 Hz), α-band (8–12 Hz), β-band (15–30 Hz), and γ-band (>30 Hz). According to neurophysiological studies, these rhythms contribute to the coordination of neuronal activity in remote brain regions (Lisman and Jensen, 2013; Fries, 2015). For instance, Canolty et al. (2006) clearly demonstrate that the low-frequency θ-rhythm modulates electrical brain activity at the high-frequency γ-band of the electrocorticogram (ECoG). Apart from the θ-band, according to Fries (2015), the low-frequency α- and β-band neuronal activity in visual cortex controls the neuronal activity in the γ-band. This means that the high-frequency spiking activity of single neurons is modulated by a low-frequency rhythm that spreads over distributed cortical regions.

The functional connectivity between cortical regions is usually measured in terms of correlation or synchronization of the recorded EEG signals for different rhythms (Lisman and Jensen, 2013; Fries, 2015). As stated in Maksimenko et al. (2017a), neuronal populations in remote brain regions interact at different frequency bands with different strengths. Recent studies (Buffalo et al., 2011; Michalareas et al., 2016) demonstrate that during the performance of visual tasks, neural populations in the visual cortex communicate at frequencies in the joint α, β (8–30 Hz) and γ (50–70 Hz) ranges. Moreover, an analysis of the functional connectivity between regions of the parieto-occipital cortex performed on the EEG sensory level reveals a different connectivity structure in separated α- and β-frequency bands, while the functional connectivity in the β-band is affected by visual information complexity (Maksimenko et al., 2018c). Along with the neuronal communication in the visual cortex, accomplishing the visual task requires communication between the remote cortical regions. For instance, during visual information processing, δ-activity in the frontal area and α-activity in the parieto-occipital area are functionally coupled and jointly guide visual perception to integrate sensory evidence with current task demands (Helfrich et al., 2017). During a sustained attention task, a long-range functional connectivity between different parts of the frontoparietal network is mediated by oscillations in the θ-band, and connectivity within these areas is subserved by γ-band oscillations (Sellers et al., 2016). The attention-related functional connectivity was also found in the frontoparietal cortex in different frequency ranges (Clayton et al., 2015; Scolari et al., 2015).

Summarizing the above discussion, we highlight the following results related to functional connectivity during the perceptual decision-making task performance.

- Accomplishing the perceptual decision-making task requires coordination of neural activity across multiple cortical areas in the frontoparietal network.
- Coordination of neuronal activity in particular regions is subserved by high-frequency rhythms, while the coordination of neural activity between remote regions relies on low-frequency oscillations.
- Functional interactions dynamically reconfigure the neuronal network structure to maintain sustained attention and avoid fatigue and distraction during task performance.
- fMRI and EEG studies evidence spontaneous fluctuations of functional connectivity correlated with the fluctuations of behavioral performance during a prolonged attention task.

Despite numerous studies, the mechanism of functional network reconfiguration underlying spontaneous behavioral fluctuations during a demanding task remains unclear. To address this issue, we consider functional connectivity in the cortical network by analyzing EEG signals in low-frequency α- and β-bands during a prolonged perceptual decision-making task which requires sustained attention.

Generally, sustained attention refers to the ability to focus on relevant stimuli with repeated presentation over extended periods. In consonance with Miodrag and Hodapp (2011), the tasks of sustained attention often involve long series of presentations of target and non-target stimuli on computer screens, to which the participants must respond to the targets and refrain from responding to the non-target stimuli. In our study, we considered a perceptual decision-making task implying a binary classification of a large number of consistently presented ambiguous visual stimuli (Necker cubes) with different degrees of ambiguity (Kornmeier et al., 2011; Maksimenko et al., 2017b; Hramov et al., 2018). In line with Denison et al. (2018), we suppose that processing each stimulus depends on the level of attention during the moment of its presentation. Since the stimuli are subsequently presented with 3–5 s pauses and each stimulus is presented for 1–1.5 s, the subject has to constantly maintain a high level of attention to successfully respond to the stimuli.

The Necker cube image classification can be considered as an example of perceptual decision-making, which is known to include two stages (sensory processing and decision-making; Mostert et al., 2015). Usually, perceptual decision-making is not viewed as a classical cognitive domain like attention or memory. At the same time, this is mostly true for near-threshold stimuli (Weisz et al., 2014) or unambiguous stimuli when the subject has to choose between two totally different stimuli. In our experiments, we use ambiguous visual stimuli, whose classification causes uncertainty in decision-making when ambiguity is high (Hramov et al., 2018). Finally, in agreement with Kornmeier et al. (2017), the Necker cube interpretation can be considered as a cognitive decision process.

According to previous research (Sehatpour et al., 2008; Michalareas et al., 2016), neuronal activity in α- and β-bands represents two stages: a sensory processing stage and a perceptual decision-making stage. During the former stage, α- and β-band activity is involved in top-down stimulus processing and subserves the neural interaction within the visual cortex (Michalareas et al., 2016). The β-band activity is also shown (Sehatpour et al., 2008) to coordinate the neuronal activity in the occipital and prefrontal areas during visual stimulus processing. During the latter stage, the β-band activity subserves the neural interactions between the anterior cingulate-insula network and the fronto-parietal network during the decision-making (Chand and Dhamala, 2016, 2017). According to an earlier review (Siegel et al., 2011), the decision accuracy correlates with the power of the frontoparietal β-band activity registered during the decision period of the trial. A wide body of literature shows that both α- and β-band activity is relevant to attention in general (i.e., not restricted to the visual stimuli processing; Linkenkaer-Hansen et al., 2004; Gola et al., 2013; Baumgarten et al., 2014). Attention modulates the prestimulus α- and β-band power (Anderson and Ding, 2011; Bauer et al., 2012; Gola et al., 2013) and affects decision accuracy. Thus, either medium or low α- and high β-band power during the prestimulus period is beneficial for sensory perception (Van Dijk et al., 2008; Gola et al., 2013). According to Hanslmayr et al. (2007), not only the power but also the prestimulus EEG phase coupling in the α- and β-bands affects visual perception performance. Namely, better performance is associated with low phase coupling in the α-band and high phase coupling in the β-band.

Thus, the visual sensory processing stage is characterized by the pronounced α- and β-band activity in the occipital cortex, whereas the decision-making stage is associated with an increase in the β-band activity across the frontoparietal cortex. Conforming to these studies, we suppose that since the neural interactions in the α- and β-bands are involved in both the sensory processing and the decision-making stages, and at the same time characterized by different spatial configurations, the functional network structure should change during the transition from one stage to another.

In this work, we analyze the reaction time (RT) defined as the time interval between the stimulus presentation and the subject's behavioral response (button pressing). We find that RT depends on the stimulus ambiguity and, more importantly, fluctuates in time, even when the ambiguity remains unchanged. Interestingly, such RT fluctuations for stimuli with similar ambiguity are not associated with mental fatigue since long RTs dominate at the beginning of the experiment. We associate these RT fluctuations with the functional network reconfiguration under stimulus classification. We suppose that RT includes two temporally separated stages: visual information processing and decision-making. These stages require the emergence of large-scale neuronal interactions within a functional cortical network. Our results reveal that neuronal interactions during the decision-making stage do not affect RT; it is almost the same regardless of the stimulus ambiguity. We hypothesize that RT is affected by the processes preceding the decision-making stage, e.g., encoding visual sensory information and extracting decision-relevant features from raw sensory information.

## 2. Materials and Methods

In this section we provide the detailed description of experimental and computational methods, including reporting on human participants, visual stimuli and timing parameters, recording instruments and characteristics, data preprocessing and connectivity analysis (Gross et al., 2013; Keil et al., 2014).

### 2.1. Participants

Twenty healthy unpaid volunteers, 11 males and 9 females, between the ages of 26 and 35 with normal or corrected-to-normal visual acuity participated in the experiments. All of them provided informed written consent before participating. The experimental studies were performed in accordance with the Declaration of Helsinki and approved by the local research Ethics Committee of the Innopolis University.

### 2.2. Visual Stimuli

**Figure 1:**
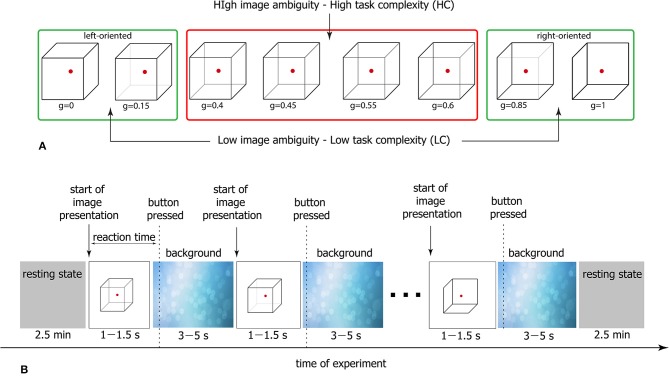
**(A)** Complete set of visual stimuli divided into two subsets according to the degree of ambiguity. Classification of cubes with high ambiguity is a high-complexity (HC) task, whereas classification of low-ambiguous cubes is a low-complexity (LC) task. **(B)** Schematic representation of the experimental protocol.

The Necker cube is a 2D image which looks like a cube with transparent faces and visible edges (Figure 1). An observer without any perception abnormalities perceives the Necker cube as a bistable 3D object due to the specific position of the inner edges. The value *g* ∈ [0, 1] defining a contrast of the three middle edges is usually used as a control parameter. It is calculated as *g* = *y*/255, where *y* is the brightness of the middle lines according to the 8-bit grayscale palette. The values *g* = 1 and *g* = 0 correspond, respectively, to 0 (black) and 255 (white) pixels' luminance of the middle lines. Each Necker cube image drawn by black and gray lines was located at the center of the computer screen on a white background. A red dot drawn at the center of the Necker cube was used to attract the subject's attention and prevent possible perception shifts due to eye movements while observing the image. To demonstrate stimuli, we used a 24” BenQ LCD monitor with a spatial resolution of 1920 × 1080 pixels and a 60-Hz refresh rate. The subjects were located at a distance of 70–80 cm from the monitor with a visual angle of ~0.25 rad. The Necker cube size on the monitor was 14.2 cm. The visual task was to classify consistently presented ambiguous Necker cubes with different *g* as left- or right-oriented (Kornmeier et al., 2011; Hramov et al., 2017).

As seen in Figure 1, the degree of ambiguity *g* indicates how difficult it is to determine a correct cube orientation. While for *g* ≈ 1 and *g* ≈ 0 the Necker cubes can easily be classified as a left- or a right-oriented one, for *g* ≈ 0.5 the classification task is more complex since we deal with a highly ambiguous image. In our experiment, we present to each subject a set of Necker cubes with *g* ∈ [0;0.15;0.4;0.45;0.55;0.6;0.85;1] divided into two subsets *g* ∈ [0.4;0.45;0.55;0.6] (high-ambiguous images) and *g* ∈ [0;0.15;0.85;1] (low-ambiguous images; Figure 1A). It is clear that the classification of the cubes belonging to the former subset is a simpler task and therefore it is considered as a low-complexity (LC) task, whereas the classification of the cubes from the latter subset requires a higher cognitive effort and hence it is referred to as a high-complexity (HC) task.

### 2.3. Different Task Conditions

**Figure 2:**
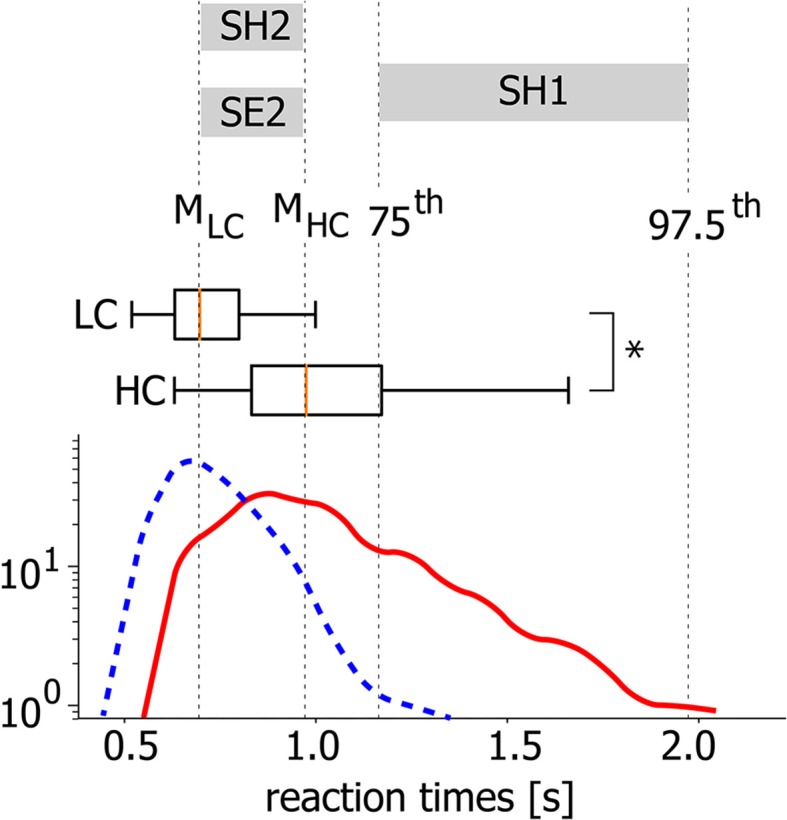
Different task conditions. Typical probability distribution functions (PDFs) of RT for HC (solid line) and LC (dashed line) tasks and corresponding box-and-whisker diagrams for a single subject (*p < 0.05 via Mann-Whitney *U*-test for 200 stimuli). *M*_*LC*_ and *M*_*HC*_ correspond to medians of the PDFs.

Generally, accomplishing an LC task takes lower RT than a HC task. At the same time, for both LC and HC tasks, RTs are distributed within a certain time interval. In Figure 2 the typical RT distributions are presented for LC and HC tasks as probability density functions (PDF) and box-and-whisker diagrams. While the statistical test evidences a significant difference between the mean values (*p < 0.05 via Mann-Whitney *U*-test for 200 stimuli), there is a time interval for which the considered PDFs are overlapped (0.6–1.1 s for this particular case). Thus, it can be supposed that along with the task complexity, neural activity can be considered as a potential candidate underlying different RTs during the accomplishing of tasks with similar complexity.

To reveal the mechanism underlying such a behavior, we consider three groups of EEG trials defined in accordance with different conditions of stimuli perception based on the relation between stimulus complexity and corresponding RT (see Figure 2).

- **SH1** condition includes trials associated with long RTs during HC task accomplishing. RTs belong to the interval between 75th and 97.5th PDF percentiles.
- **SH2** condition includes trials associated with short RTs during HC task accomplishing. RTs belong to the interval between PDF medians.
- **SE2** condition includes trials associated with RTs similar to SH2 during LC task accomplishing. RTs belong to the interval between PDF medians.

The described choice of visual perception conditions aims at the analysis of neuronal mechanisms underlying changes in RT related to stimulus complexity (SH1 vs. SE2) and regardless of it (SH1 vs. SH2). After the process selection, each group contains 23–37 trials. To keep the number of trials constant across different conditions and subjects, we consider *K* = 20 randomly selected trials in each group.

### 2.4. Experimental Procedure

The participants were not subjected to a training session before the main experiment. Each subject participated in a 40-min experiment, during which the Necker cubes with different *g* randomly selected from the whole set of stimuli (Figure 1A) were presented about 50 times. The structure of the experimental session is schematically shown in Figure 1B. The participants were instructed to press either the left key with their left hand or the right key with their right hand to indicate the first impression on the orientation of each successively presented cube. In the consecutive presentation of bistable images, previously demonstrated cubes could affect the perception of subsequent cubes. We refer this phenomenon to as memory effect. For example, if the subject observes several left-oriented cubes in a row, then his/her perception will stabilize the left-oriented cube, even if the following cube is right-oriented. To reduce the memory effect (Leopold et al., 2002), each stimulus was presented for a time interval randomly selected from the [1–1.5] s range. A random variation of the control parameter *g* was applied to prevent the perception stabilization. Lastly, to draw away the observer's attention and make the perception of the succeeding cube independent of the previous one, different abstract pictures were exhibited for about 3–5 s between subsequent demonstrations of the Necker cubes.

### 2.5. EEG Recording

The EEG signals were recorded by 31 sensors (see Table 1) with two reference electrodes A1 and A2 on the earlobes and a ground electrode N just above the forehead. To record EEG data, we used cup adhesive Ag/AgCl electrodes placed on the “Tien-20” paste (Weaver and Company, Colorado, USA). Immediately before the experiments started, we performed all necessary procedures to increase skin conductivity and reduce its resistance using the abrasive “NuPrep” gel (Weaver and Company, Colorado, USA). The impedance was monitored after the electrodes were installed and measured throughout the experiments. Usually, the impedance values varied within a 2–5 kΩ interval. The electroencephalograph “Encephalan-EEG-19/26” (Medicom MTD company, Taganrog, Russian Federation) with multiple EEG channels and a two-button input device (keypad) was used for amplification and analog-to-digital conversion of the EEG signals. This device possessed the registration certificate of the Federal Service for Supervision in Health Care No. FCP 2007/00124 of 07.11.2014 and the European Certificate CE 538571 of the British Standards Institute (BSI). The raw EEG signals were filtered by a band-pass filter with cut-off points at 1 Hz (HP) and 100 Hz (LP) and by a 50-Hz notch filter by an embedded hardware-software data acquisition complex.

**Table 1:** Experimental parameters.

**Parameter**	**Value**
Number of participants	20
Duration of stimulus presentation	randomly chosen between 1 and 1.5 s, step size 0.016 s
Interval between stimulus presentations	randomly chosen between 3 and 5 s, step size 0.064 s
Number of stimulus configurations	8
Total number of presented stimuli	400
Total duration of the experimental session	40 min
Recording time of the resting state EEG	5 min
Location of EEG scalp electrodes	International 10-10 system (31 channels)
EEG recording sampling rate	250 Hz
EEG recording filter	1–100 Hz (bandpass), 50 Hz (notch) filters
Considered EEG frequency bands	α-waves (8–12 Hz), β-waves (15–30 Hz)
Experimental conditions	SE2, SH2, SH1
Number of trials per condition	20

Recorded EEG signals presented in proper physical units (millivolts) were segmented into a set of 4-s trials, where each trial, associated with a single presentation of the Necker cube, included a 1-s interval before and a 3-s interval after the cube demonstration, due to specific needs of connectivity analysis methods. To reduce muscular artifacts, the participants were asked to take a pose which excluded excessive tension of neck muscles. However, a number of artifacts still appeared in the EEG data. Artifacts caused by eye movement, muscle activity and cardiac rhythm were corrected with the method based on empirical mode decomposition (Maksimenko et al., 2018b). Artifacts were considered as properly removed if their EEG amplitude after filtration dropped below 30% of the initial value. Trials where artifacts were not properly removed were excluded. The overall number of trials used for every subject was about 350 out of the initial 400.

### 2.6. Functional Connectivity Analysis

We investigate functional connectivity based on the analysis of spectral power correlations in α- and β-frequency bands using the recurrence measure of dependence. As reported by Hipp et al. (2012) and Siegel et al. (2012), amplitude or spectral power correlations may be informative indicators of large-scale neuronal interactions during cognitive activity despite the fact that amplitude and phase correlation emerge independently from each other. Thus, inference of functional connectivity between two EEG signals is performed according to the following steps.

**Figure 3:**
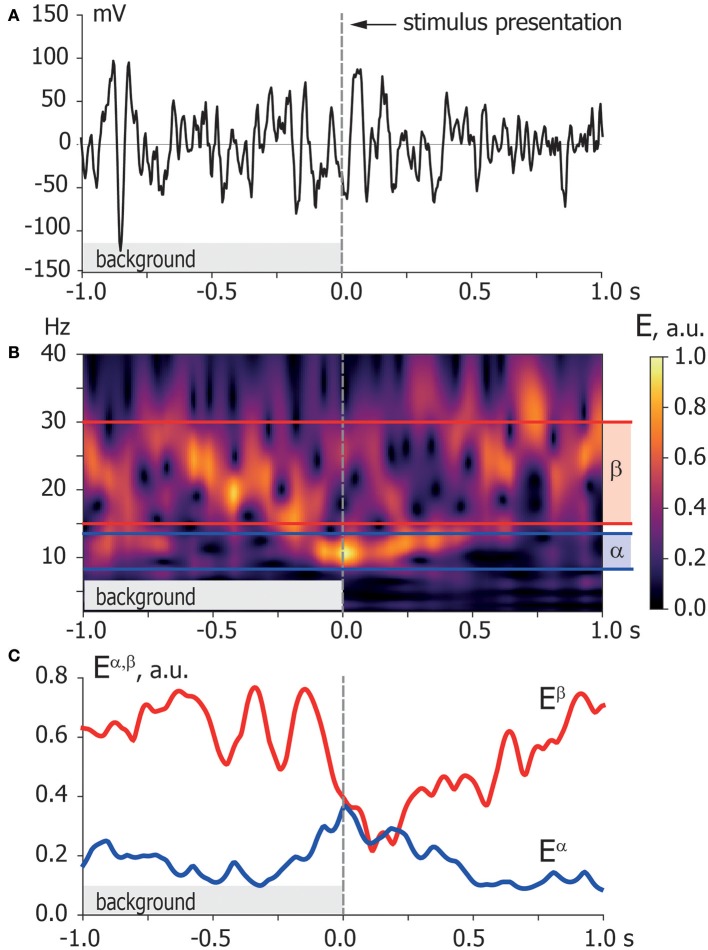
Time-frequency representation. **(A)** Typical EEG trial recorded from O1 sensor during background activity [–1,0] s and stimulus processing [0,1] s. The moment of stimulus presentation is indicated with a vertical dashed line. **(B)** Time-frequency representation of the EEG trial via wavelet transform with highlighted α and β frequency bands. **(C)** Spectral power of α and β oscillations *E*^α, β^ extracted from time-frequency representation of EEG trial according to Equation (2).

**Step 1. Wavelet transform**. To provide functional connectivity analysis in terms of amplitude correlation, we extract time-varying spectral power in α- and β-bands from recorded EEG signals. We provide the time-frequency representations of EEG signals via a continuous wavelet transform (Hramov et al., 2015) (see Figure 3):

$$\begin{array}{rcl} W\left(f,t_{0}\right) & = & \sqrt{f}\int_{-\infty }^{+\infty }x\left(t\right)\psi^{*}\left(f\left(t-t_{0}\right)\right)dt, \end{array}$$

(1) $$\begin{array}{rcl} \psi \left(\eta \right) & = & \frac{1}{\sqrt[{4}]{\pi }}e^{j\omega_{0}\eta }e^{-\frac{\eta^{2}}{2}}, \end{array}$$

where *x*(*t*) is a raw EEG signal, ψ(η) is a Morlet complex function, and ω_0_ = 2π is the wavelet central frequency. Time evolutions of spectral powers *E*^α^(*t*) and *E*^β^(*t*) calculated as

$$\begin{array}{rcl} E^{\alpha }\left(t\right) & = & \int_{f\in f_{\alpha }}|W\left(f,t\right)|df, \end{array}$$

(2) $$\begin{array}{rcl} E^{\beta }\left(t\right) & = & \int_{f\in f_{\beta }}|W\left(f,t\right)|df \end{array}$$

characterize dynamics of oscillatory neuronal activity in the *f*_α_ (8–12 Hz) and *f*_β_ (15–30 Hz) frequency bands, respectively. We use these time series for the functional connectivity analysis in terms of spectral power correlation.

Schoffelen and Gross (2009) stated that restoration of functional connectivity from a surface-level EEG signal could be problematic due to a field spread effect, resulting in unexpectedly high correlation between the signals of neighboring electrodes. Having analyzed Pearson's correlation of *E*^α, β^(*t*) for all combinations of neighboring electrodes in a resting state, we obtained poor influence of the field spread effect in these particular frequency bands (median correlation coefficient ρ < 0.6 for both α- and β-bands).

To verify that power correlations are not influenced by the systematic power differences between conditions, we compared averaged power spectra at each electrode during stimulus processing (1 s after stimulus presentation) using two-factorial ANOVAs with the condition (SH1 vs. SH2 vs. SE2) and electrodes as within-subject factors with Bonferroni correction for multiple comparisons. Repeated measures ANOVA demonstrates the absence of the significant difference of the wavelet spectral power between conditions in both α-band [*F*_(2, 38)_ = 1.092, *p* = 0.346, SE2 (0.148 ± 0.011 SE), SH2 (0.153 ± 0.011 SE), and SH1 (0.152 ± 0.011 SE)] and β-band [*F*_(2, 38)_ = 0.179, *p* = 0.837, SE2 (0.488 ± 0.018 SE), SH2 (0.489± 0.016 SE), and SH1 (0.491 ± 0.017 SE)].

**Step 2. Recurrence-based measure of dependence**. The recurrence-based approach for inference functional links from the time series (Goswami et al., 2013; Ramos et al., 2017) inspired by the concepts of non-linear dynamics, is a suitable method to explore synchronization and directed non-linear dependencies in the functional network of the brain cortex, especially while considering relatively short trials. Recurrence is a fundamental property possessed by natural processes and means that a considered process recurs to the neighborhood of its earlier state. Recurrence plot (RP), a visualization of a recurrence process, is a powerful tool for system analysis using time series (see a comprehensive review of Marwan et al., 2007). The comparison of recurrence structures of two processes using joint recurrence provides information on the interrelation between them. The idea standing behind this statement was suggested by Romano et al. (2005), who stated that two processes are related by a functional dependence (in case of generalized or lag synchronization; Boccaletti et al., 2014) if they have similar recurrence plots. Based on this statement, Goswami et al. (2013) developed a connectivity measure called *recurrence-based measure of dependence* (RMD). This measure indicates “non-independence” and its direction obtained from given time series introducing a lag in a possible driving process. In all, RMD determines the presence or absence of a causal relation in a pair of processes in terms of establishment of a functional relationship (linear or non-linear) between them. Recently, this recurrent-based approach was applied to analyze functional connectivity in short biological and climatic time series (Goswami et al., 2013; Ouyang et al., 2013; Builes-Jaramillo et al., 2018; Maksimenko et al., 2018a).

To calculate RMD values, we analyze the time series of the spectral power in α- and β-bands Equation (2) obtained via wavelet transform of the original EEG data. In order to estimate a statistically significant increase (or decrease) in the coupling strength for each interchannel link during stimulus processing against a preceding baseline, we calculate corresponding RMD values for *K* = 20 presented stimuli.

Let a subject observe *K* visual stimuli and $E_{xy}^{k}\left(t\right)$ be a time series of a spectral power in a particular frequency band, recorded from *x* and *y* EEG channels during *k*th stimulus processing, calculated by Equation (2). Then, RMD indicating the level of interdependence between rhythmic neuronal activity in *x* and *y* EEG channels, is defined as

(3) $$\begin{array}{rcl} RMD{|}_{xy}^{k} & = & \log_{2}\left(\frac{1}{N}\overset{N}{\underset{i=1}{\sum }}RMD_{i}{|}_{xy}^{k}\right), \end{array}$$

(4) $$\begin{array}{rcl} RMD_{i}{|}_{xy}^{k} & = & \frac{P\left(E_{x}^{k}\left(t_{i}\right),E_{y}^{k}\left(t_{i}\right)\right)}{P\left(E_{x}^{k}\left(t_{i}\right)\right)P\left(E_{y}^{k}\left(t_{i}\right)\right)}, \end{array}$$

where *N* is a length of the time series, $P\left(E_{x,y}^{k}=E_{x,y}^{k}\left(t_{i}\right)\right)$ is a probability for $E_{x,y}^{k}$ to take the value $E_{x,y}^{k}\left(t_{i}\right)$, and $P\left(E_{x}^{k}\left(t_{i}\right),E_{y}^{k}\left(t_{i}\right)\right)=P\left(E_{x}^{k}=E_{x}^{k}\left(t_{i}\right)\right)P\left(E_{y}^{k}=E_{y}^{k}\left(t_{i}\right)\right)$ is a joint probability that $E_{x}^{k}=E_{x}^{k}\left(t_{i}\right)$ at the same time, where $E_{y}^{k}=E_{y}^{k}\left(t_{i}\right)$. These probabilities are determined using recurrence matrix calculations (Marwan et al., 2007) as follows

(5) $$\begin{array}{rcl} P\left(E_{x,y}^{k}\left(t_{i}\right)\right) & = & \frac{1}{N}\overset{N}{\underset{j=1}{\sum }}{\text{R}}_{x,y}^{k}\left(i,j\right), \end{array}$$

(6) $$\begin{array}{rcl} P\left(E_{x}^{k}\left(t_{i}\right),E_{y}^{k}\left(t_{i}\right)\right) & = & \frac{1}{N}\overset{N}{\underset{j=1}{\sum }}{\text{JR}}^{k}\left(i,j\right), \end{array}$$

(7) $$\begin{array}{rcl} {\text{JR}}^{k} & = & {\text{R}}_{x}^{k}\left(i,j\right){\text{R}}_{y}^{k}\left(i,j\right), \end{array}$$

where ${\text{R}}_{x,y}^{k}\left(i,j\right)$ are recurrence matrices of $E_{x}^{k}\left(t\right)$ and $E_{y}^{k}\left(t\right)$ time series and **JR**^*k*^(*i, j*) is their joint recurrence matrix. The recurrence matrix calculation was performed using the Python package *pyunicorn* (Donges et al., 2015).

To examine the direction of the non-linear dependence between *x* and *y*, we introduce an appropriate time lag τ in the definition of *RMD* given by Equations (3) and (4). Let us suppose that *x* drives *y*, then Equations (3) and (4) can be rewritten as follows

(8) $$\begin{array}{rcl} RMD\left(\tau \right){|}_{xy}^{k} & = & \log_{2}\left(\frac{1}{N^{\prime }}\overset{N^{\prime }}{\underset{i=1}{\sum }}RMD_{i}{|}_{xy}^{k}\left(\tau \right)\right), \end{array}$$

(9) $$\begin{array}{rcl} RMD_{i}\left(\tau \right){|}_{xy}^{k} & = & \frac{P\left(E_{x}^{k}\left(t_{i}\right),E_{y}^{k}\left(t_{i}+\tau \right)\right)}{P\left(E_{x}^{k}\left(t_{i}\right)\right)P\left(E_{y}^{k}\left(t_{i}+\tau \right)\right)}, \end{array}$$

where *N*′ = *N*−τ and $E_{y}^{k}\left(t_{i}+\tau \right)$ is a time series of $E_{y}^{k}$ shifted τ units with respect to $E_{x}^{k}$. The dependence of RMD on lag τ has a local maximum $\tau^{*}{|}_{xy}^{k}=\text{argmax}\left[RMD{|}_{xy}^{k}\left(\tau \right)\right]$ which indicates the relevant time lag between interacting processes, while its sign determines the direction of a functional link; *x* drives *y* if $\tau^{*}{|}_{xy}^{k}>0$ and *y* drives *x* otherwise. Also, $RMD^{*}{|}_{xy}^{k}=RMD{|}_{xy}^{k}\left(\tau^{*}{|}_{xy}^{k}\right)$ represents a relevant measure of the coupling strength between *x* and *y*.

**Step 3. Single-subject statistics**. The aim of the statistical analysis is the quantification of reliability of changes in the functional network structure obtained from single-subject data (Gross et al., 2013). First, we test the significance of a functional link between *x* and *y* by the pairwise comparison of samples $RMD_{b}{|}_{xy}=\left\{RMD_{b}^{*}{|}_{xy}^{1},RMD_{b}^{*}{|}_{xy}^{2},\ldots ,RMD_{b}^{*}{|}_{xy}^{K}\right\}$ and $RMD_{t}{|}_{xy}=\left\{RMD_{t}^{*}{|}_{xy}^{1},RMD_{t}^{*}{|}_{xy}^{2},\ldots ,RMD_{t}^{*}{|}_{xy}^{K}\right\}$ composed of RMD^*^ values during background (1 s prior task accomplishment) and task-related activity via *t*-test for related samples (*K* = 20 stimuli). To attack the multiple comparison problem (MCP), we use a non-parametric method implying estimation of distributions with maximal statistics across tests by permutation with 2,000 iterations according to Maris and Oostenveld (2007). Finally, the corrected value of α-level is used to define significant changes in functional links. When *p* < α, changes in the coupling strength are supposed to be significant and the link is classified according to the value of *S* = sign[median(*RMD*_*t*_|_*xy*_)−median(*RMD*_*b*_|_*xy*_)], i.e., *S* > 0 for the link with increasing coupling strength and (*S* > 0) with decreasing coupling strength.

**Field spread effect**. The connectivity analysis from multichannel EEG or MEG data is usually problematic due to volume conduction and field spread effect (Schoffelen and Gross, 2009). This phenomenon consists in a spatial spread of electromagnetic fields, thus one recording channel or sensor might pick up the activity of multiple neuronal sources (Cohen, 2017). The field spread effect, which causes spurious functional links, should obviously be taken into account during functional connectivity analysis from EEG or MEG data. The strategies for avoiding the field spread effect were described by Bastos and Schoffelen (2016). We suppose that the proposed method follows some of them. First, our measure reveals significant changes in the emergence of functional links during the stimulus-related process in contrast to pre-stimulus brain dynamics associated with the observation of the abstract image. Thus, the comparison of these two states based on MCP corrected statistical test should effectively reduce the number of spurious estimates. Second, our method uses a connectivity metric that analyzes lagged dependencies and does not consider instantaneous (zero-phase) interactions which usually contain most of the false correlations caused by the field spread effect. Moreover, the provided connectivity analysis is based on the wavelet spectral-power time-series, where phase dynamics of the raw EEG data is excluded. Therefore, the effects of false zero-phase correlations emerging due to a common source are reduced as well.

**Graph metrics**. To describe functional network evolution during visual stimulus processing, we use the following graph metrics: the ratio *R* between the number of increasing and decreasing links and outgoing node degree *D*_*i*_. We suppose that a functional connectivity network is described by two matrices, **M**_*inc*_ and **M**_*dec*_, containing links with increasing and decreasing coupling strengths, respectively. Then, the ratio between the number of increasing and decreasing links is defined as

(10) $$\begin{array}{r} R=\frac{\sum_{i=1}^{N_{s}}\sum_{j=1}^{N_{s}}{\text{M}}_{inc}\left(i,j\right)}{\sum_{i=1}^{N_{s}}\sum_{j=1}^{N_{s}}{\text{M}}_{dec}\left(i,j\right)}, \end{array}$$

where *N*_*s*_ is a number of EEG sensors. The outgoing node degree *D*_*i*_ determines the number of increasing links outgoing from the *i*^*th*^ EEG sensor and defined as

(11) $$\begin{array}{r} D_{i}=\overset{N_{s}}{\underset{j=1}{\sum }}{\text{M}}_{inc}\left(i,j\right). \end{array}$$

Next, the averaged outgoing node degree *D* over a certain brain area can also be used to quantify functional network properties in a group of participants. Let a brain area *A* be composed of *N*_*a*_ EEG sensors {*x*_1_, *x*_2_, …, *x*_*N*_*a*__}. Then, the averaged outgoing node degree is

(12) $$\begin{array}{r} D=\frac{1}{N_{a}}\underset{x_{i}\in A}{\sum }D_{i}. \end{array}$$

**Across-subjects statistics**. To provide statistical analysis across participants we compare the above-described graph metrics, which characterize integral properties of the single-subject functional connectivity. In particular, we use repeated measures ANOVA to perform a multi-factor analysis. We consider averaged degree *D* as a dependent variable with brain areas (O, P, Cp, C, Fc, F, Fp), experimental conditions (SE2, SH2, SH1), and time as within-subject factors.

**Figure 4:**
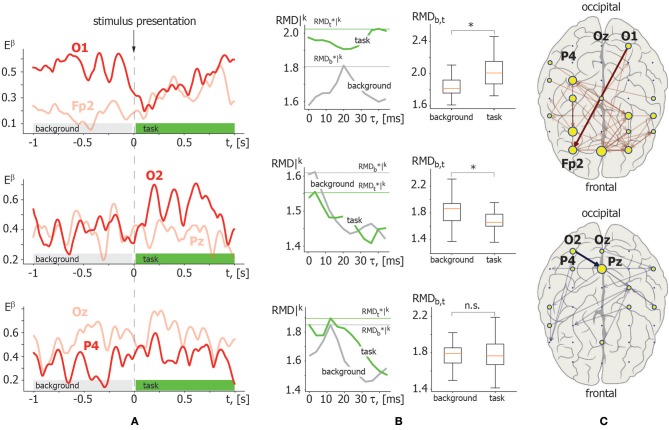
Illustration of recurrence-based approach for functional links inference. **(A)** β-band spectral power *E*^β^(*t*) calculated for the following pair of EEG channels in a single trial: O1–Fp2 (upper panel); O2–Pz (middle panel); Oz–P4 (lower panel). The moment of stimulus presentation is indicated with a vertical dashed line. **(B)** Left column: *RMD*|^*k*^(τ) dependence for considered pairs of *E*^β^(*t*) trials in background (gray) and visual perception (green) activity. Maximal values $RMD_{b,t}^{*}{|}^{k}$ are indicated by horizontal lines. Right column: results of pairwise comparison of maximal RMD values collected over *K* = 20 trials for background and task-related brain activity via *t*-test for related samples. Here, ^*^ indicates significance level of *p* < α via *t*-test for related samples corrected for MCP by non-parametric permutation test. **(C)** β-band functional networks containing links with increasing (upper panel) and decreasing (lower panel) coupling strength related with visual stimuli processing. Bold arrows indicate links selected for recurrence-based method demonstration.

**Illustrative example**. Figure 4 demonstrates the application of the above described RMD measure. Let us consider β-band functional connectivity with three pairs of EEG signals: O1–Fp2, O2–Pz, and Oz–P4. Figure 4A illustrates the evolution of β-band spectral power *E*^β^(*t*) for these EEG signals during single visual stimulus processing. The left column in Figure 4B shows the results of RMD(τ) calculation based on the recurrence analysis of *E*^β^(*t*) for each pair of EEG channels presented in Figure 4A. The graphs of RMD(τ) are calculated for both background and visual perception brain activities to estimate changes in the functional coupling strength and direction. The right column in Figure 4B illustrates the results of pairwise comparison of the functional coupling strength in background and stimulus perception phases collected over *K* = 20 trials via *t*-test for related samples with MCP correction. The example demonstrates that functional coupling in the Oz–P4 link does not change significantly. By contrast, temporal correlation increases in the O1–Fp2 pair and decreases in the O2–Pz pair during visual perception. Finally, typical functional connectivity structures restored for a single participant are presented in Figure 4C. The upper panel shows the functional connectivity network composed of links with increasing coupling strength, while the lower panel shows the network structure with decreasing links.

## 3. Results

### 3.1. Reaction Times and Task Complexity

**Figure 5:**
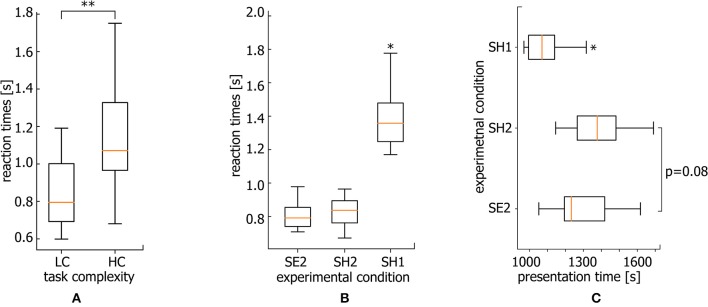
Reaction times. **(A)** Median RT for HC and LC tasks averaged over all subjects (**p < 0.01 via Wilcoxon signed-rank test, *n* = 20 subjects). **(B)** Median RT for SE2, SH2, and SH1 conditions averaged over all subjects (*p < 0.05, via repeated measures ANOVA with Bonferroni correction, *n* = 20 subjects). **(C)** Median presentation times over the course of the experiment for stimuli belonging to SE2, SH2, and SH1 conditions averaged over all subjects (^*^p < 0.05 via repeated measure ANOVA with Bonferroni correction, *n* = 20 subjects).

The performance of a more complicated task as known to require longer RT. In particular, the classification of highly ambiguous Necker cubes (HC task) takes longer RT than less ambiguous right- or left-oriented cubes (LC task). Following Rousselet and Wilcox (2019), we use median to describe the central tendency of RT for each subject. As a result, the median RT reaches 1.1 s for HC tasks while the median RT for LC tasks is only 0.8 s (for comparison see Figure 5A, *p < 0.05 via Wilcoxon signed-rank test).

Figure 5B shows the median RTs of SH1, SH2, and SE2 trials via box-and-whiskers diagrams reflecting the full distribution of the data (Weissgerber et al., 2015; Rousselet et al., 2016). The repeated measures ANOVA with Greenhouse-Geisser correction reveal a significant difference between RT within conditions [*F*_(1.03, 19.57)_ = 25.57, *p* < 0.001]. In addition, *post-hoc* comparison using paired sample *t*-test with Bonferroni correction reveals a significant difference for SH1 vs. SE2 (ΔRT = 0.563 s ± 0.08 SE) (*p* < 0.001, *t* = −5.208, *df* = 19) and SH1 vs. SH2 (ΔRT = 0.527 s ± 0.077 SE) conditions (*p* < 0.001, *t* = −4.997, *df* = 19). At the same time, SE2 and SH2 conditions are characterized by a comparable RT (ΔRT = 0.039 s ± 0.012 SE). While the significantly larger RT for SH1 with respect to SE2 condition is intuitively clear, and can be explained by higher complexity of the stimuli, the significant difference between SH1 and SH2 piques the interest. One expects that an increase in RT can be caused by mental fatigue due to a long duration of the task performance. However, longer RTs dominate at the beginning of the experiment.

Median times of SH1, SH2, and SE2 stimuli presentation during the experimental session are shown in Figure 5C. The repeated measures ANOVA reveal a significant difference between these three conditions [*F*_(2.0, 38.0)_ = 11.43, *p* < 0.001]. Specifically, the Bonferroni corrected pairwise comparison shows that SH1 condition stimuli are mostly presented earlier as compared with SE2 (*p* = 0.018, *t* = −3.094, *df* = 19) and SH2 (*p* = 0.004, *t* = −3.792, *df* = 19) conditions. Thus, we conclude that the RT growth is not a consequence of mental fatigue, since RTs are larger at the beginning of the experiment.

### 3.2. Functional Network Reconfiguration

To reveal neural mechanisms underlying the observed behavioral results, we analyze the functional network reconfiguration during the visual stimuli processing in each of three conditions. First, we determine functional links whose strength exhibits a significant change with respect to the value obtained before the Necker cube presentation (see Methods).

**Figure 6:**
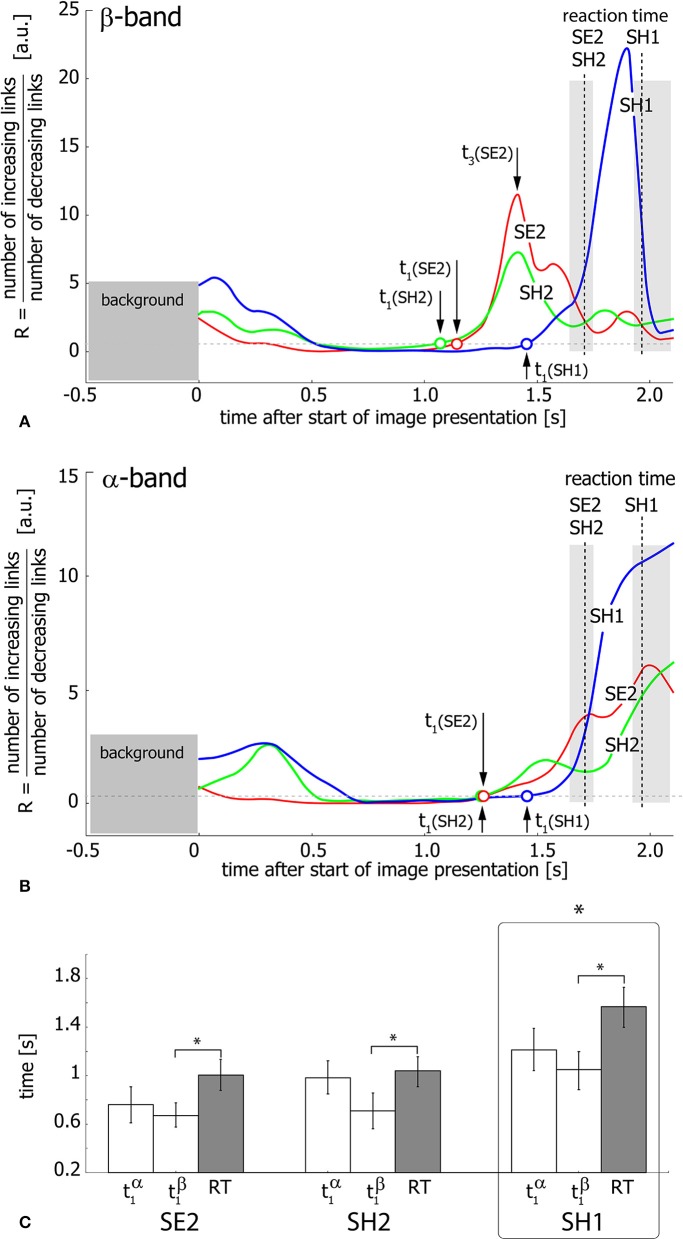
Functional network reconfiguration. **(A,B)** Ratio *R* of the number of increasing functional links to the number of decreasing links in β- and α-frequency bands. The arrows indicate the moments of time *t*_1_(SE2, SE2, SH1) when *R* exceeds the level of *R* = 1 shown by the dashed horizontal line. *t*_3_(SE2) is a time moment when *R* reaches the maximal value for SE2 condition. The vertical dashed lines indicate median RT and the shaded area around these lines illustrates RT distributions in the 25th–75th percentile. **(C)** Median values of *t*_1_ and median RT for each of the three conditions (SE2, SH2, SH1) for the group of 20 subjects. The data are shown as group mean ± SE (^*^p < 0.05 via repeated measures ANOVA and *post-hoc* pairwise comparison with Bonferroni correction).

Figure 6 shows the ratio *R* of the number of increasing links to the number of decreasing links during visual stimulus perception and processing in the β (Figure 6A) and α (Figure 6B) bands. Different curves in the graphs correspond to SH1 (blue), SH2 (green), and SE2 (red) experimental conditions and dashed vertical lines indicate median RTs inside the shaded areas corresponding to the 25th–75th percentile. As seen from Figures 6A,B, the ratio *R* exhibits growth, reflecting the prevalence of increasing functional links before the subject's behavioral response. This means the development of densely-connected spatially-distributed functional networks in both considered frequency bands. The moments of time *t*_1_(SH1, SH2, SE2) when *R* crosses the unity level, are considered as starting points of this process for SH1, SH2, and SE2 experimental conditions, respectively.

To relate the functional connectivity structure formation in α- and β-bands to the behavioral response, the extracted moments of time $t_{1}^{\alpha ,\beta }$(SH1, SH2, SE2) are compared with RT via repeated measure ANOVA (Figure 6C). Different experimental conditions (SH1, SH2, SE2) and time moments ($t_{1}^{\alpha }$, $t_{1}^{\beta }$, RT) are considered as within-subject factors. As a result, ANOVA shows a significant difference between conditions [*F*_(2, 38)_ = 15.826, *p* < 0.001]. Moreover, ANOVA with Greenhouse-Geisser correction shows a significant difference between $t_{1}^{\alpha ,\beta }$ and RT within each condition [*F*_(1.07, 20.33)_ = 8.461, *p* < 0.05]. *Post-hoc* tests using Bonferroni correction reveal that SH1 significantly differs from both SH2 (*p* = 0.0024, *t* = −3.98, *df* = 19) and SE2 (*p* = 0.0015, *t* = −4.184, *df* = 19), while the difference between conditions SH2 and SE2 is insignificant (*p* = 0.228, *t* = −1.875, *df* = 19). In addition, *post-hoc* analysis based on the paired samples *t*-test with Bonferroni correction reveals a significant (*p* = 0.023, *t* = −2.982, *df* = 19) difference between $t_{1}^{\beta }$ (0.81 s ± 0.11 SE) and RT (1.204 s ± 0.10 SE), while the difference between $t_{1}^{\alpha }$ (0.983 s ± 0.1 SE) and RT is insignificant (*p* = 0.101, *t* = −2.289, *df* = 19) for all conditions. Finally, $t_{1}^{\alpha }$ is shown to significantly exceed $t_{1}^{\beta }$ across conditions (*p* = 0.003, *t* = 3.843, *df* = 19). Importantly, according to the repeated measures ANOVA, the time interval between $t_{1}^{\beta }$ and RT is (0.394 s ± 0.115 SE) and differs insignificantly between conditions [*F*_(2, 38)_ = 2.606, *p* = 0.087].

Thus, for all experimental conditions, the network structure formation in the β-band starts earlier than that in the α-band and about 0.394 s before the behavioral response. Furthermore, in the α-band, the starting point of the functional network development does not differ significantly from the subject's reaction time. Taken together, it can be supposed that the evolution of the functional connectivity structure in both β- and α-frequency bands is temporally distinguished. Thus, cognitive decision-making processes lead to the formation of a new network structure in the β-band, but not in the α-band.

### 3.3. Spatio-Temporal Features of the Functional Network

Here, we focus on functional connectivity in the β-band, whose structure, as was shown above, starts to form about 0.394 s before the actual behavioral response. We consider the evolution of the functional connectivity in the β-band on the time interval of [*t*_1_, *t*_3_], where *t*_1_ is a starting point of the network structure formation, *t*_3_ is associated with the maximal value of *R* (see Figure 6), and *t*_2_ = (*t*_3_−*t*_1_)/2 is chosen to analyze the transient network topology.

The *R* values reflecting the connectivity evolution in the β-band are analyzed via repeated measures ANOVA under experimental conditions (SE2, SH2, SH1) at time moments (*t*_1_, *t*_2_, *t*_3_) as within-subject factors. As a result, ANOVA with Greenhaus-Geiser correction shows a significant difference between *R* values at the time moments [*F*_(1.005, 19.095)_ = 7.143, *p* < 0.05]. *Post-hoc* analysis using paired sample *t*-test with Bonferroni correction shows a significant increase in *R*(*t*_3_) with respect to *R*(*t*_1_) (*p* = 0.042, *t* = 2.706, *df* = 19) and *R*(*t*_2_) (*p* = 0.048, *t* = 2.644, *df* = 19). Thus, we verify that *R* increases during the time interval [*t*_1_, *t*_3_] indicating the formation of a spatially extended cortical network in the β-band. Finally, according to repeated measures ANOVA, no significant difference between *t*_3_ and RT was found for all conditions [*F*_(1, 19)_ = 2.88, *p* = 0.106]. Thus, we conclude that the functional network in the β-band forms at the moment of the subject's behavioral response.

**Figure 7:**
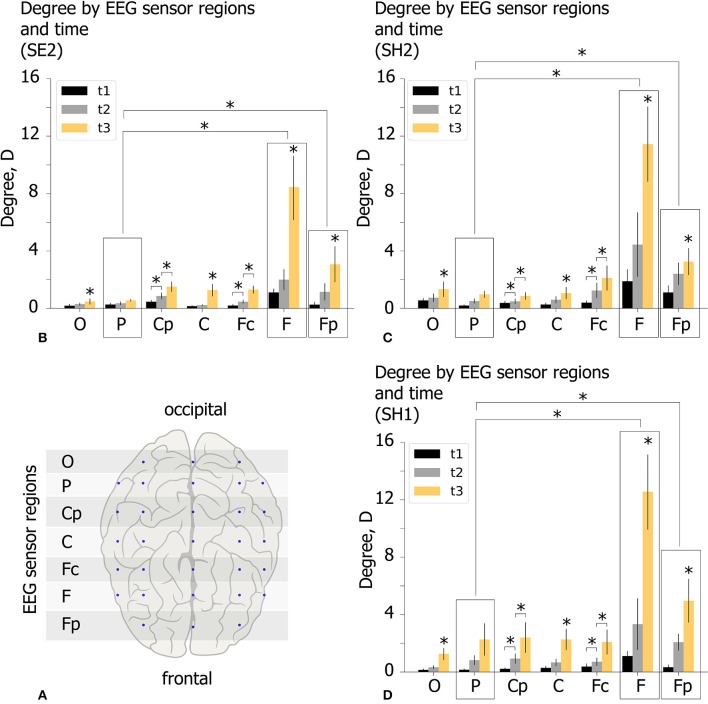
Properties of decision-making functional connectivity in β-band. **(A)** Schematic illustration of selected EEG sensor regions. **(B–D)** The degree *D* (mean ± SE) vs. EEG sensor region (horizontal axis) and time (different color) for different experimental conditions (SE2, SH2, and SH1) (^*^p < 0.05 via repeated measures ANOVA with Bonferroni correction).

The spatio-temporal features of the functional network in the β-band are analyzed for experimental conditions SH1, SH2, and SE2 at time moments (*t*_1_, *t*_2_, *t*_3_) by considering the topology of increasing links. For each participant we estimate the mean degree of outgoing links *D* for seven EEG sensor regions shown by the horizontal shaded lines in Figure 7A. These regions are defined by means of equal distance in order to parcellate the electrode along the longitudinal brain axis. Each region includes EEG sensors on both hemispheres. Lateral effects are not considered since each condition includes equal proportions of the left- and right-oriented Necker cubes. The value of *D* characterizes the mean number of outgoing links from each region. Consequently, a high *D* value reflects a leading role of a particular EEG sensor region in the cognitive process. The obtained *D* values (group mean) are shown in Figures 7B–D for different experimental conditions (SE2, SH2, and SH1) by the colored histogram. Each EEG sensor region is characterized by three bars of different color, where the color indicates the time moment according to the figure legend.

The obtained *D* values were analyzed via repeated measures ANOVA. The experimental condition (SE2, SH2, SH1), time (*t*_1, 2, 3_) and EEG sensor region (O–Fp) were considered as within-subject factors. ANOVA with a Greenhouse-Geisser correction shows that *D* differs significantly between time points [*F*_(1.08, 20.52)_ = 14.758, *p* < 0.01], EEG sensor regions [*F*_(1.15, 21.85)_ = 10.503, *p* < 0.01], and different time*area conditions [*F*_(1.34, 25.46)_ = 8.237, *p* < 0.05]. Importantly, *D* changes insignificantly between different experimental conditions [*F*_(2, 38)_ = 0.48, *p* = 0.618]. These results indicate that *D* in different EEG sensor regions changes differently over time, but the way it varies over time was the same regardless of experimental conditions.

*Post-hoc* analysis using paired samples *t*-test with Bonferroni correction reveals that *D* changes significantly between all time moments: *D*_*t*_1__<*D*_*t*_2__(*p* = 0.007, *t* = −3.512, *df* = 19), *D*_*t*_2__ < *D*_*t*_3__,((*p* = 0.0033, *t* = −3.755, *df* = 19), *D*_*t*_1__ < *D*_*t*_3__(*p* = 0.003, *t* = −3.924, *df* = 19) and between EEG sensor regions: *D*^P^ < *D*^F^(*p* = 0.047, *t* = −4.37, *df* = 19), *D*^P^ < *D*^Fp^(*p* = 0.045, *t* = −4.442, *df* = 19). To analyze the time*region interaction effect, we run *post-hoc* ANOVA for each EEG sensor region separately with experimental condition and time as within-subject factors. A significant within-subject time effect is found for all EEG sensor regions except area P. The further pairwise comparison using paired samples *t*-test with Bonferroni correction reveals a significant increase in *D*_*t*_2__ with respect to *D*_*t*_1__ for regions **Cp** (*D*_*t*_1__ = 0.35 s ± 0.06 SE, *D*_*t*_2__ = 0.76 s ± 0.14 SE, *p* = 0.031, *t* = 2.831, *df* = 19), **Fc** (*D*_*t*_1__ = 0.32 s ± 0.06 SE, *D*_*t*_2__ = 0.87 s ± 0.16 SE, *p* = 0.046, *t* = 2.664, *df* = 19)and a significant increase in *D*_*t*_3__ with respect to *D*_*t*_2__ for regions **O** (*D*_*t*_2__ = 0.45 s ± 0.1 SE, *D*_*t*_3__ = 1.02 s ± 0.19 SE, *p* = 0.021.*t* = 3.021, *df* = 19), **Cp** (*D*_*t*_2__ = 0.76 s ± 0.14 SE, *D*_*t*_3__ = 1.59 s ± 0.36 SE, *p* = 0.028, *t* = 2.901, *df* = 19), **C** (*D*_*t*_2__ = 0.49 s ± 0.11 SE, *D*_*t*_3__ = 1.53 s ± 0.22 SE, *p* = 0.001, *t* = 4.365, *df* = 19), **Fc** (*D*_*t*_2__ = 0.87 s ± 0.17 SE, *D*_*t*_3__ = 1.83 s ± 0.31 SE, *p* = 0.002, *t* = 4.079, *df* = 19), **F** (*D*_*t*_2__ = 3.26 s ± 0.88 SE, *D*_*t*_3__ = 10.81 s ± 2.57 SE, *p* = 0.013, *t* = 3.237, *df* = 19), and **Fp** (*D*_*t*_2__ = 1.87 s ± 0.37 SE, *D*_*t*_3__ = 3.76 s ± 0.7 SE, *p* = 0.031, *t* = 2.856, *df* = 19).

Summarizing the results of the statistical analysis, we conclude that spatio-temporal evolution of the β-band functional brain network in the time interval of [*t*_1_, *t*_3_] takes place in a similar way regardless of experimental conditions. During the growth of the network structure from *t*_1_ to *t*_3_, each EEG sensor region except region P exhibits a significant increase in the number of outgoing links *D*, especially pronounced in the regions F and Fp.

## Discussion

We considered a perceptual decision-making task consisting in the classification of the ambiguous visual stimuli (Necker cubes) according to its interpretation as left-or right-oriented. Having analyzed the response time (RT), which the subject spent for the Necker cube classification, we have found that stimuli with high ambiguity usually required ~0.3 s longer RT than stimuli with low ambiguity. We also discovered that RT was not constant for stimuli with the same ambiguity. In particular, RT to the same highly ambiguous stimulus was larger at the beginning of the experiment than at the end (1.4 vs. 0.8 s). This allows us to conclude that the observed changes in RT are not related to mental fatigue, which is known to cause an increase in the RT (Langner et al., 2010). In turn, the decreasing RT in the course of the experiment can be associated with the training effect. The role of training in the performance of the perceptual decision-making tasks was demonstrated by Yang et al. (2014). The authors suggest that the training could improve the efficiency of high-level visual processing, which therefore would provide less ambiguous sensory information to the decision-related brain networks. While in Yang et al. (2014) the training period lasted for 3 days, our results suggest that the training effect could already be notable even within a 40-min session.

To investigate the neuronal activity which is supposed to stand behind the obtained behavioral results, we considered the evolution of functional connectivity on EEG sensor level as spectral power correlation separately in α and β frequency bands. Our results demonstrate that in both bands the functional connectivity exhibits the formation of a spatially-extended network. The structure formation in the β-band starts earlier than that in the α-band and earlier than RT. In the α-band, the starting point of the network formation does not differ significantly from the RT. This allows us to conclude that the evolution of the functional connectivity structure in the β- and α-frequency bands is temporally distinguished. A detailed analysis of the spatial properties of the β-band network reveals that frontal areas are characterized by a higher degree of outgoing links with respect to the parietal area.

It is known that perceptual decision-making implies encoding sensory information, accumulating this sensory input over time and planning an ensuing motor action, and the neuronal populations in sensory, parietal and frontal cortices are involved in different stages of this process (Hanks and Summerfield, 2017). It was also shown that the perception and decision-making stages are temporally dissociated. The sensory information processing is limited to an early time window (0.13–0.35 s) and associated with occipital areas, whereas decision-related processing is increasingly pronounced over time and involves parietal and frontal areas (Mostert et al., 2015). According to a previous review (Siegel et al., 2011), perceptual decisions are mediated by oscillatory interactions in the β-band in the large-scale frontoparietal network during the decision period of the trial. In addition, Rahnev et al. (2016) also report a critical role of the frontal cortex in the control of perceptual decision-making. Taking these results into account, we suppose that the observed reorganization of the β-band functional network with the highly involved frontal zone confirms that neuronal processes captured in the [*t*_1_, *t*_3_] interval are associated with the decision-making stage.

Finally, our results demonstrate that the time lag between $t_{1}^{\beta }$ and RT takes around 0.39 s for all stimuli, indicating that the duration of the decision-making state does not affect the overall RT. Based on this result, we assume that changes in RT are related to the mechanisms of the neuronal activity preceding the decision-making stage. In particular, it can be supposed that the earlier sensory processing stage is affected by the quality of the visual sensory information. If stimulus ambiguity is increased, more sensory evidence is required to make a decision; therefore, the sensory processing stage can spend more time. As reported by Siegel et al. (2006, 2011), stimulus features can affect the brain activity related to the encoding of sensory evidence. Using the motion discrimination task, the authors demonstrate that this process is associated with occipital γ-band oscillations, and the motion strength (the strength of the evidence) affects the amplitude of these oscillations. Moreover, Philiastides and Sajda (2005) found the relation between the duration of the sensory evidence accumulation process and the strength of visual evidence. Using a face vs. car categorization task they demonstrated that as the evidence for faces vs. cars decreases, the stimulus processing time increases. In the case of the Necker cube classification, an increase in the cube ambiguity can be associated with a decrease in the evidence for left-oriented vs. right-oriented cubes. Therefore, the processing of highly ambiguous stimuli takes a longer time than processing stimuli with low ambiguity.

Lastly, the changes in RT can also be related to the processes underlying the extraction of decision-relevant information of the stimulus from raw sensory information acquiring during visual perception (Wyart et al., 2012). As noted in Siegel et al. (2008), the brain can optimize this process by selecting only those features of sensory evidence that are relevant for the particular task. The top-down selection of sensory evidence is commonly referred to as *selective attention*. Having considered the selective attention in the context of perceptual decision-making, Siegel et al. (2011) suggest that this process is subserved by a long-range oscillatory synchronization between frontoparietal regions and early sensory processing stages and mediates the selection of sensory information features required for the perceptual decision. We suppose that this process can be a potential reason for decreasing RT in the course of the experiment. Since in our perceptual decision-making task the presented Necker cubes are distinguished from each other by the contrast of some inner edges, the brain is likely to start using this particular feature of visual information to make a decision according to the Necker cube interpretation.

## 4. Conclusion

In this paper, we have analyzed the reaction time (RT) and functional neuronal interactions in α- and β-frequency bands during the perceptual decision-making task of ambiguous visual stimuli classification.

Behavioral analysis revealed an increase in RT as the stimulus ambiguity is increased. It is important that RT fluctuates among stimuli even when their ambiguity remains unchanged. The observed RT fluctuations are not associated with mental fatigue, because longer RTs dominate at the beginning of the experiment. We suppose that the decreasing RT throughout the experiment can be caused by the training effect, which improves the efficiency of high-level visual processing and therefore provides less ambiguous sensory information to the decision-related brain networks.

Functional connectivity analysis evidenced that the subject's behavioral response is preceded by the emergence of a large-scale functional network in the β-frequency band with the pronounced driving role of frontal cortical areas, which we associate with the decision-making network. Both the structural properties of this network and the time required for its development are independent of the stimulus ambiguity and do not affect RT.

To conclude, we suppose that RT fluctuations are related to the processes preceding the decision-making stage, e.g., encoding visual sensory information and extracting decision-relevant features from raw sensory information. According to the literature, the duration of these stages can be affected by the quality of the sensory evidence and selective attention, implying the brain's ability to select only those features of sensory evidence which are needed for a particular task.

## Data Availability Statement

The datasets generated for this study are available on request to the corresponding author.

## Ethics Statement

The studies involving human participants were reviewed and approved by Ethics Committee of the Innopolis University. The patients/participants provided their written informed consent to participate in this study.

## Author Contributions

VM, NF, AH, AP, and JK conceived the study. VG and AR performed experiments. VM provided behavioral analysis and interpreted the results. NF conducted network analysis. VM, NF, AH, and AP wrote the manuscript.

### Conflict of Interest

The authors declare that the research was conducted in the absence of any commercial or financial relationships that could be construed as a potential conflict of interest.
